# Influence of mammographic density and compressed breast thickness on true mammographic sensitivity: a cohort study

**DOI:** 10.1038/s41598-023-41356-2

**Published:** 2023-08-30

**Authors:** Rickard Strandberg, Maya Illipse, Kamila Czene, Per Hall, Keith Humphreys

**Affiliations:** 1 Department of Medical Epidemiology and Biostatistics, Karolinska Institutet, Stockholm, Sweden; 2 Swedish eScience Research Centre (SeRC), Karolinska Institutet, Stockholm, Sweden

**Keywords:** Breast cancer, Statistics

## Abstract

Understanding the detectability of breast cancer using mammography is important when considering nation-wide screening programmes. Although the role of imaging settings on image quality has been studied extensively, their role in detectability of cancer at a population level is less well studied. We wish to quantify the association between mammographic screening sensitivity and various imaging parameters. Using a novel approach applied to a population-based breast cancer screening cohort, we specifically focus on sensitivity as defined in the classical diagnostic testing literature, as opposed to the screen-detected cancer rate, which is often used as a measure of sensitivity for monitoring and evaluating breast cancer screening. We use a natural history approach to model the presence and size of latent tumors at risk of detection at mammography screening, and the screening sensitivity is modeled as a logistic function of tumor size. With this approach we study the influence of compressed breast thickness, x-ray exposure, and compression pressure, in addition to (percent) breast density, on the screening test sensitivity. When adjusting for all screening parameters in addition to latent tumor size, we find that percent breast density and compressed breast thickness are statistically significant factors for the detectability of breast cancer. A change in breast density from 6.6 to 33.5% (the inter-quartile range) reduced the odds of detection by 61% (95% CI 48–71). Similarly, a change in compressed breast thickness from 46 to 66 mm reduced the odds by 42% (95% CI 21–57). The true sensitivity of mammography, defined as the probability that an examination leads to a positive result if a tumour is present in the breast, is associated with compressed breast thickness after accounting for mammographic density and tumour size. This can be used to guide studies of setups aimed at improving lesion detection. Compressed breast thickness—in addition to breast density—should be considered when assigning complementary screening modalities and personalized screening intervals.

## Introduction

Breast cancer (BC) is the most common cancer type affecting women^1,2^. In Europe, full-field digital mammography is the frontline imaging tool for early detection, and nation-wide mammography screening programmes have been introduced in many countries^3^. In Sweden, women between the ages of 40 and 74 are invited to attend every 18–24 months^4^. At screening, each breast, in turn, is pressed between a detector and a paddle, and x-rayed from two different angles. The breast is compressed in order to fix the breast in place, spread the dense breast tissue, reduce scatter radiation, and lower the required radiation dose. The resulting four images are then assessed by radiologists for BC findings.

It is important to determine the extent to which screening is able to correctly identify the presence and absence of BC, so that its findings encourage appropriate decision making. The adequacy and usefulness of a screening test is usually determined by the sensitivity (and specificity) of the test. In this paper we focus on sensitivity, specifically mammographic sensitivity. There are multiple reasons why a lesion can be missed on a mammogram, which can broadly categorized into technical errors, related to the quality of the image; perceptual errors, including masking and poor conspicuity of the lesion; and cognitive errors, related to misinterpretation and misclassification of the findings^5^.

For monitoring mammography screening performance, sensitivity is commonly estimated as the proportion of BCs that are detected in close proximity to a screening, and this “sensitivity” is usually calculated using 1–2 year follow-ups for cancer detection^6,7^. These screen-detected cancers are in contrast to interval cancers—BC detected symptomatically some time after a screening or between screening rounds—which are assumed to represent the cancers that were present at the previous screening but missed^8,9^. While this assumption is true for many of the interval cancers, some particularly fast-growing tumors will not have been present or not have been of a detectable tumor size (so called *true* interval cancers). Also, regular screening could mean that slow-growing tumors were present at multiple screenings, but only contribute towards the most recent screening interval^10^. These two scenarios mean that this definition of sensitivity can be considered as being biased and diverges from the classical definition of sensitivity in the statistical literature of diagnostic testing. It does not take the tumor size, nor the tumor growth rate, into account.

Whereas most studies report one constant estimate for screening sensitivity, some researchers have developed a modelling approach for estimating the screening sensitivity of mammography as a function of tumor size^11,12^, where sensitivity is part of a larger natural history model applied to population-based breast cancer screening data. In these natural history models, the latent tumor sizes at prior (negative) screens are (probabilistically) back-calculated. Logistic functions represent the probability of detecting a BC tumor, given its (latent) size at the time of the screening. This way of estimating screening sensitivity is therefore closer to the statistical definition of a test sensitivity^13^. To differentiate this definition of screening sensitivity, we will refer to this as the *Screening Test Sensitivity* (STS).

Many factors intricately affect mammographic sensitivity. Percent breast density (the proportion of fibroglandular radio-dense tissue in the breast) is known to be an important masking factor, since both tumors and dense tissue appear white on mammograms^14–16^. Mammographic density, in general, is considered to be the most important masking factor. Other factors that influence the appearance and quality of a mammogram include the distribution of the dense tissue, imaging techniques such as position settings and image processing conditions^9,17^. Here we study the associations of such factors with STS, in particular, total x-ray exposure, compression pressure, and compressed breast thickness, the latter being the distance between the two mammography plates after the breast is compressed. The role of such factors on image quality and radiation dose has been well studied^18^. The relationship between compression pressure and ‘sensitivity’ (as defined in terms of the ratio of screening to interval cancers) has also been studied using population-based data^19–21^, but as explained above, our approach to this is different. Studies^19,20^, like ours, use measurements of compression pressure based on standard automatic exposure control (AEC) settings. Moreover, we are not aware of population-based studies of the role of x-ray exposure and compressed breast thickness in mammography sensitivity. Studies have tried to identify good levels of compression and compressed breast thickness^19,22,23^ with varying focus.

Since mammographic density plays such an important role in masking tumors, studies exploring the role of other screening parameters need to account for density, as we do here. It is important to highlight that the composition of the breast changes with age. Several studies have demonstrated that breast density declines substantially with increasing age, mainly over the menopausal transition^24^, and reaches a plateau around the age of 65^25^. This age-related difference in breast composition has been shown to be concomitant with the increase of sensitivity of mammography with age^9,26^. We take this into account in our study by using multiple (longitudinal) measurements of the density and related acquisition parameters.

In addition to biological changes to breast density, the estimated density based on the mammogram is prone to measurement variability based on the placement of the breast, and other imaging settings. Studies with short-term repeated imaging have shown that this variability exists both for computer-aided PD estimation^27–29^, and for radiologist assessed density categorization^27,28,30^.

## Materials and methods

### Data

We utilise data from the Karolinska Mammography Project for Risk Prediction of Breast Cancer (KARMA)^31^. KARMA is a Swedish prospective mammography screening cohort. During the period January 2011 to March 2013, women attending mammography screening at any one of four selected hospitals in Sweden were invited to participate. A total of 70,877 women accepted, filled in an extensive web-based questionnaire, and gave blood samples. Both raw and digitally processed mammograms are stored. Cases of BC are identified through a Swedish national quality register.

In this study we focus on the following screening parameters:

- Percent mammographic density (PD), the proportion of radio-dense tissue on the mammogram (0–1),
- Compressed breast thickness (CBT), the distance between the paddles when the image is taken (cm),
- Exposure (EXP), the total x-ray dose, i.e. x-ray current times the exposure time (mAs),
- Compression pressure (CP), calculated as the compression force divided by the measured compressed breast area (N/$\hbox {cm}^2$),
- Total breast volume (TBV), the measured compressed area on the image times the CPT.

The PD was measured using STRATUS^32^, and the other screening parameters were extracted from the DICOM information tag of each image. These variables are taken from the mediolateral oblique (MLO) view, and were retrieved from each individual screening, and for each woman. Women with missing information on any of these parameters on any of the screenings were excluded from the study.

It is conventional when studying the effect of PD on breast cancer risk to use the contralateral (non-cancer) breast for the breast cancer cases. This is done so that the presence of the tumor does not add to the measurement of PD. However, when studying the detectability of breast cancer and screening parameters, the tumor side is the relevant side. Furthermore, our approach is based on there being latent undiagnosed tumors among some of the censored women (i.e. women that were not diagnosed with BC during follow-up). We do not know which women have latent tumors, nor do we know which sides such tumors are in. As our solution to this, we took the average between the left and right breasts, for each variable. We however also conducted a sensitivity analysis where we used the contralateral breast for the cases and a randomly selected side for the censored women.

In the present study, we excluded women with a BC diagnosis prior to joining KARMA. Of the BC cases, we included only the women with invasive BC that had a recorded date of diagnosis, mode of detection (whether the BC was detected through mammography screening or symptoms), and primary tumor size.

**Figure 1:**
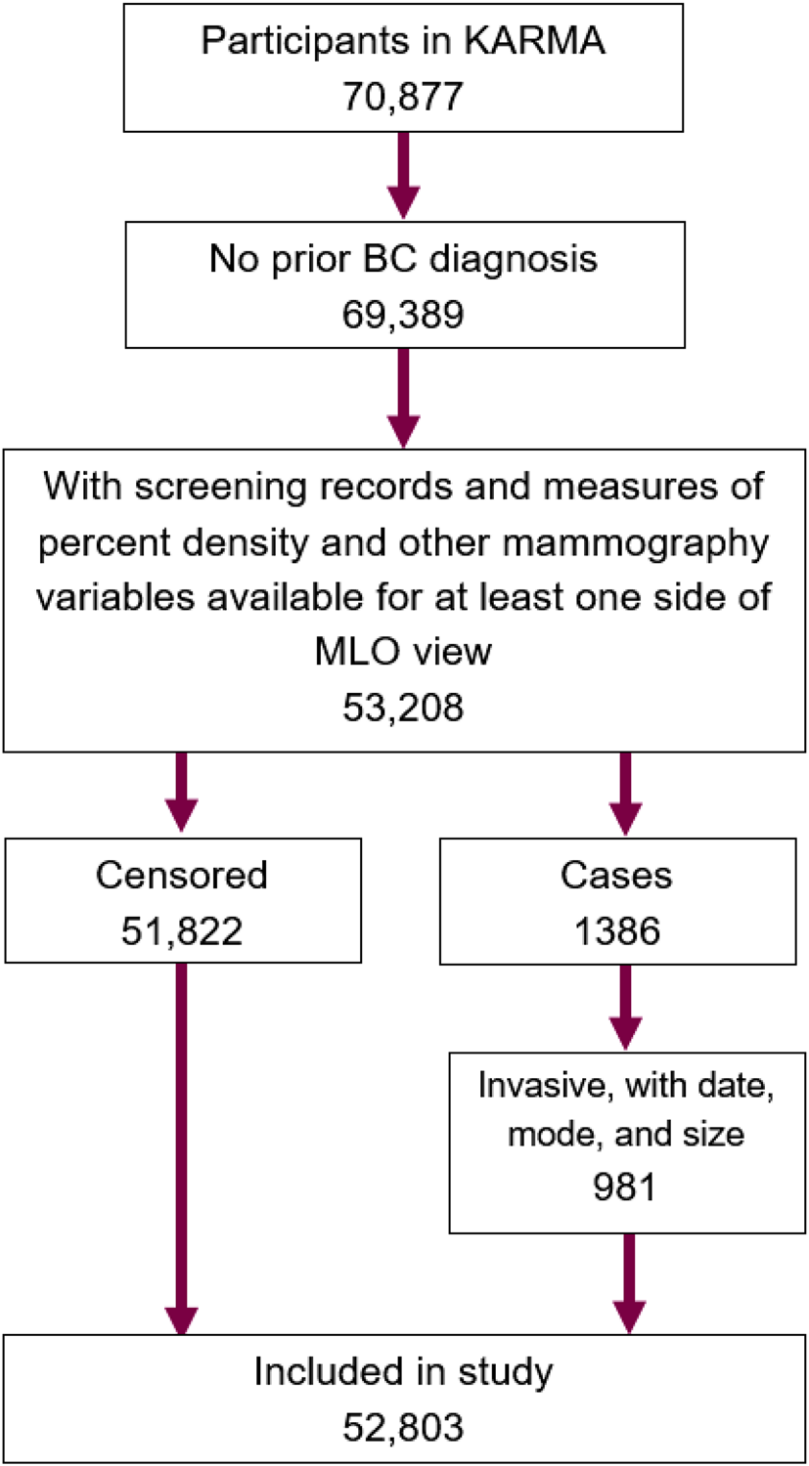
Flowchart describing the selection of the data.

Out of the 70,877 total women recruited, our study includes 52,803 women, of which 981 women were diagnosed with invasive breast cancer within the study period, which ended on 2018-02-28. In total, data from 163,053 screening occasions was included. See Fig. 1 for a summary of the data selection process.

### Statistical methods

We use a continuous growth natural history model to jointly model the time from a woman’s birth to the detection of an invasive breast cancer tumor, and tumour size at detection. This is done in a prospective population-based cohort (where the majority of women are free from BC diagnosis at end of follow-up, but nonetheless contribute to the model estimation). The natural history model has been described in detail in a previous study^33^, but for self-containment a summary is included here and in the Supplementary Material.

The natural history model can be separated into four different sub-models:

1. The carcinogenesis of the tumor, which we refer to as the age at (tumor) onset. This is a latent process and never observed. It uses the Moolgavkar-Venson-Knudson two-stage model^34^ and is determined by the three parameters $A, B, \delta$.
2. The growth of the tumor, which is assumed to be exponential with an inverse growth rate randomly drawn from a gamma distribution. This distribution is determined by the two parameters $\mu , \phi$.
3. The time until breast cancer symptoms emerge and the tumor is symptomatically detected. The continuous hazard of symptomatic detection is assumed to be proportional to the latent tumor volume such that, as it grows, it is more likely to be detected. The parameter $\eta _0$ determines the proportionality. In this study, the hazard rate is also adjusted for the total breast volume (TBV).
4. The probability that a tumor is prematurely detected should the woman attend a mammography screening. This sub-model is the primary focus of this study, and is defined as follows:

If a woman attends mammography screening before a tumor is symptomatically detected, there is opportunity to detect it early. We assume that the screening test sensitivity (STS) for the mammography screening undertaken at age $\omega$ follows a logistic function of the latent tumor diameter $D(\omega )$ at the time of the screening, i.e.

$$\begin{aligned} STS(\omega ) = \text {logit}\left( \beta _0 + \beta _s D(\omega )\right) = \frac{\text {exp}\left( \beta _0 + \beta _s \cdot D(\omega ) \right) }{1 + \text {exp}\left( \beta _0 + \beta _s \cdot D(\omega ) \right) }, \end{aligned}$$

for parameters $\beta _0, \beta _s$. The STS can be extended to depend on other factors than just tumor size. In this study, we include the possible dependence on PD, CBT, EXP, CP. The STS function is then

$$\begin{aligned} STS(\omega ) = \text {logit}\left( \beta _0 + \beta _s D(\omega ) + \beta _1[PD] + \beta _2[CBT] + \beta _3[EXP]+\beta _4[CP] \right) . \end{aligned}$$

None of the four processes in the model are directly observable with the available data. But the individual data on each woman’s age at diagnosis, tumor size at diagnosis, and mode by which the tumor was detected (symptomatically or through screening) can be used to infer their functions and distributions on a population level. This is done by conditioning backwards in time to hypothetical times of onset. For each possible age at onset, the probability of experiencing the observed outcome is calculated. For more details, see the Supplementary Material or Strandberg & Humphreys^33^.

### Ethics approval and consent to participate

The study was conducted in accordance with the Declaration of Helsinki, and approved by the Regional Ethical Review Board in Stockholm (Dnr 2010/958-31/1). Informed consent was obtained from all subjects involved in the study.

## Results

We present the key characteristics of the study population in Table 1. Of the 51 320 women included (*Materials and Methods; Data*), 640 had BC detected at one of their screenings, and 320 had BC detected symptomatically outside of, or between, screenings. The median tumor size was noticeably larger for the symptomatic cases (17 mm) than for the screen-detected (13 mm), and symptomatic cases were detected at younger ages on average (median 59 years vs. 63 years). Screen-detected women had—on average compared to symptomatic women—larger breast volume and compressed breast thickness, but lower percent density and compression pressure. These findings can be statistically confirmed by two-sample t-tests. The average total exposure was not different (a two-sample t-test gave a *p*-value of 0.23).

**Table 1:** Descriptive comparison of the variables under study, based on the three types of outcome (screen-detected breast cancer, symptomatically detected breast cancer, or censored at the end of follow-up).

	Outcome		
	Screen-detected	Symptomatic	Censored
Number of women	641	320	50 359
Age, years (median & quartiles)	63 (54, 69)	59 (50, 69)	60 (52, 69)
Tumor size, mm (median & quartiles)	13 (9, 19)	17 (12, 23)	–
Total breast volume, $\hbox {cm}^3$ (median & quartiles)	915 (636, 1262)	686 (455, 1137)	827 (546, 1176)
Percent density, % (median & quartiles)	16.1 (6.7, 29.7)	29.5 (16.0, 47.4)	17.2 (6.6, 33.5)
compressed breast thickness, cm (median & quartiles)	5.8 (4.5, 6.7)	5.3 (4.4, 6.3)	5.6 (4.6, 6.6)
Exposure, mAs (median & quartiles)	16.5 (12.0, 42.5)	16.5 (12.0, 43.0)	15.0 (11.0, 42.5)
Compression Pressure, N/$\hbox {cm}^2$ (median & quartiles)	0.71 (0.56, 0.90)	0.78 (0.60, 1.02)	0.73 (0.57, 0.94)

**Figure 2:**
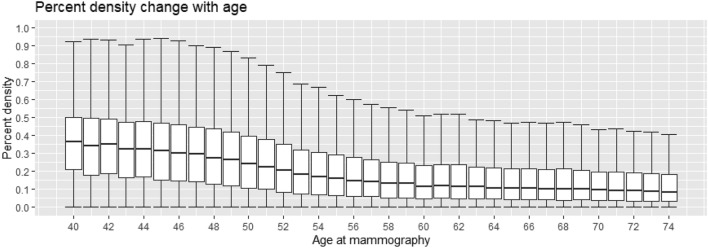
Measured percent density by age at mammography screening.

In Fig. 2 we present box plots of measured PD by age. Overall, PD reduces with age. The median PD of women aged 40–45 is 0.33 with an inter-quartile range of 0.31, while women older than 65 have median PD 0.10 with inter-quartile range of 0.17. In addition, the 95th percentile reduces from 0.69 to 0.43 between these age groups. While not visible in the figure, the 5th percentile also reduces from 0.035 to 0.007.

We started our model-based analysis by including all factors described in *Statistical Methods*; the model included TBV in the symptomatic detection rate, and PD, CBT, EXP, and CP in the logistic STS function. The maximum likelihood estimates (including 95% confidence intervals (CI)) of the model parameters are displayed in Table 2. The first six parameters are the ones related to the STS, and the rest are part of the other three submodels. To more easily compare the effects of the factors, the analysis was repeated where the model covariates were first scaled (by subtracting the sample mean and dividing by the sample standard deviation). The estimated coefficients from the scaled analysis, which represent the effect of increasing each covariate by one standard deviation, are also included in the table. PD has the largest effect, followed by CBT. CP and EXP have high *p*-values and relatively small effects.

To make the rest of the analysis more concise, we removed CP and EXP from the STS model (based on their small estimated effects and non-significant *p*-values). We also note that a likelihood ratio test comparing models with and without these two factors yielded a *p*-value of 0.45. The parameter estimates of the re-fitted/selected model is presented in Table 3. For comparison, and to highlight the significance of CBT for screening sensitivity, we also fit a version of the model with only PD in the STS (and TBV in the symptomatic detection rate). Estimates of the parameters in the STS model are (95% CI) ${\hat{\beta }}_0 = -4.88 (-5.28, -4.49), {\hat{\beta }}_s=0.51 (0.45, 0.56), {\hat{\beta }}_{PD}=-2.50 (-3.41, -1.60)$.

**Table 2:** Parameter estimates for the full model with symptomatic detection dependent on TBV, and screen-detection dependent on PD, CBT, exposure (EXP), and compression pressure (CP).

	Estimate	95% CI	Scaled	*p*-value
STS				
$\beta _0$	$-$2.948	($-$4.184, $-$1.711)	$-$5.484	
$\beta _s$	0.522	(0.461, 0.582)		
PD	$-$3.522	($-$4.620, $-$2.425)	$-$0.660	$<0.001$
CBT	$-$0.272	($-$0.423, $-$0.121)	$-$0.340	$<0.001$
CP	$-$0.135	($-$0.760, 0.490)	$-$0.052	0.672
EXP	$-$0.004	($-$0.010, 0.003)	$-$0.093	0.240
Other				
A	$-$0.084	($-$0.057, $-$0.123)		
B	0.001	(0.001, 0.002)		
$\delta$	0.068	(0.033, 0.137)		
$\mu$	0.805	(0.646, 1.002)		
$\phi$	0.468	(0.380, 0.577)		
$\eta _0$	$-$7.464	($-$7.737, $-$7.192)	$-$8.779	
TBV	$-$0.001	($-$0.002, $-$0.001)	$-$0.425	$<0.001$

**Table 3:** Parameter estimates for the final selected model with symptomatic detection dependent on TBV, and screen-detection dependent on PD and CBT.

	Estimate	95% CI	Scaled	*p*-value
STS				
$\beta _0$	$-$3.173	($-$4.068, $-$2.279)	$-$5.474	
$\beta _s$	0.513	(0.456, 0.570)		
PD	$-$3.623	($-$4.714, $-$2.532)	$-$0.669	$<0.001$
CBT	$-$0.263	($-$0.396, $-$0.131)	$-$0.330	$<0.001$
Other				
A	$-$0.078	($-$0.041, $-$0.148)		
B	0.001	(0.001, 0.003)		
$\delta$	0.076	(0.024, 0.239)		
$\mu$	0.837	(0.674, 1.039)		
$\phi$	0.487	(0.398, 0.596)		
$\eta _0$	$-$7.469	($-$7.740, $-$7.197)	$-$8.781	
TBV	$-$0.001	($-$0.002, $-$0.001)	$-$0.427	$<0.001$

**Figure 3:**
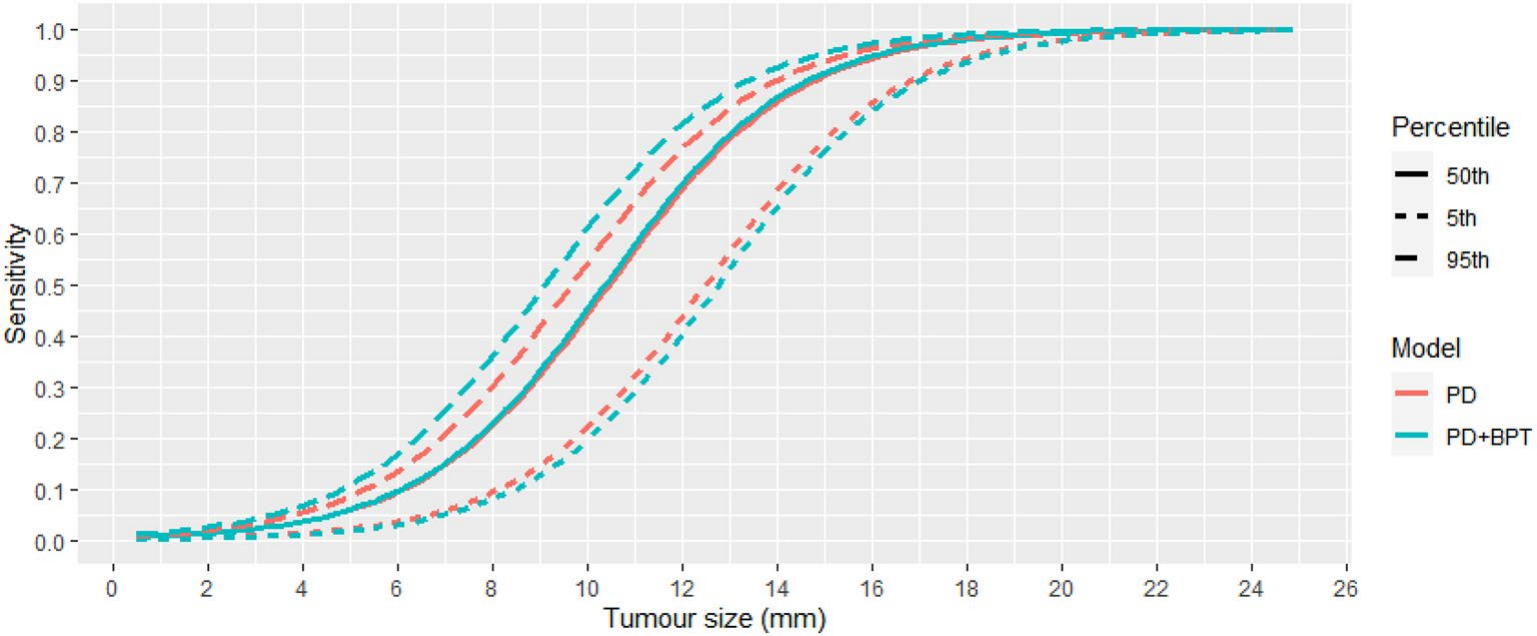
The estimated screening test sensitivity function (sub-model 4) as a function of tumor size, for different versions of the model. Quantiles of sensitivity are from the estimated sensitivity functions of each screen in the data.

Since a main finding is that CBT is associated with STS, after accounting for PD, we decided to graphically illustrate it’s contribution. We do this in Fig. 3. We took the pairs of PD and CBT values for each of the 163,053 screenings and calculated the STS for a range of tumor sizes. For each tumor size, we then plotted the 5th, 50th, and 95th percentiles of STS. We did this for two versions of the model: the (selected) model with PD and CBT (estimates in Table 3), and the model with only PD. Adding CBT helps to separate the women with high and low STS, particularly those with high STS. The maximum difference between the 5th and 95th percentiles for STS was 0.44 (or 44 percentage points) for the model with PD and CBT, and 0.34 (or 34 percentage points) for the model with only PD.

**Figure 4:**
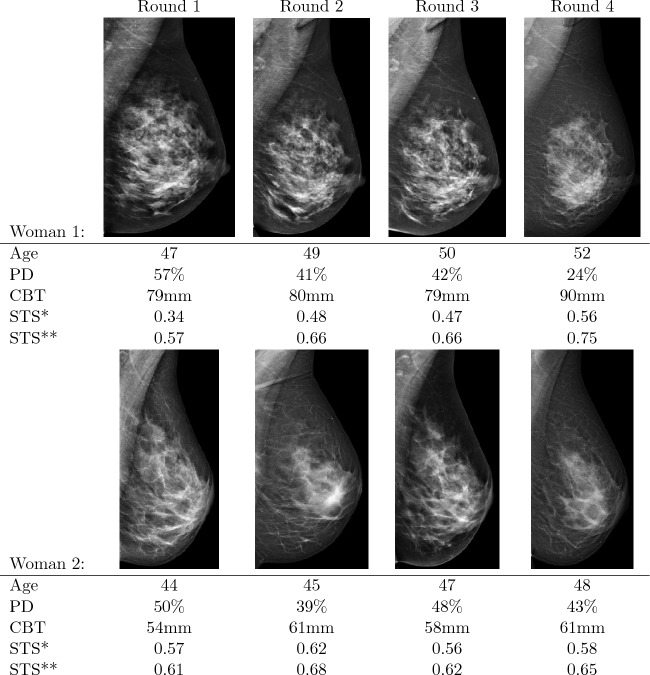
Example of two women’s sequential mammograms over 5 years, including age and the measured values of percent density (PD) and compressed breast thickness (CBT). *Estimated screening test sensitivity (STS) for a hypothetical 13 mm tumor when accounting for PD and CBT. **Estimated STS for a hypothetical 13 mm tumor when accounting only for PD.

**Figure 5:**
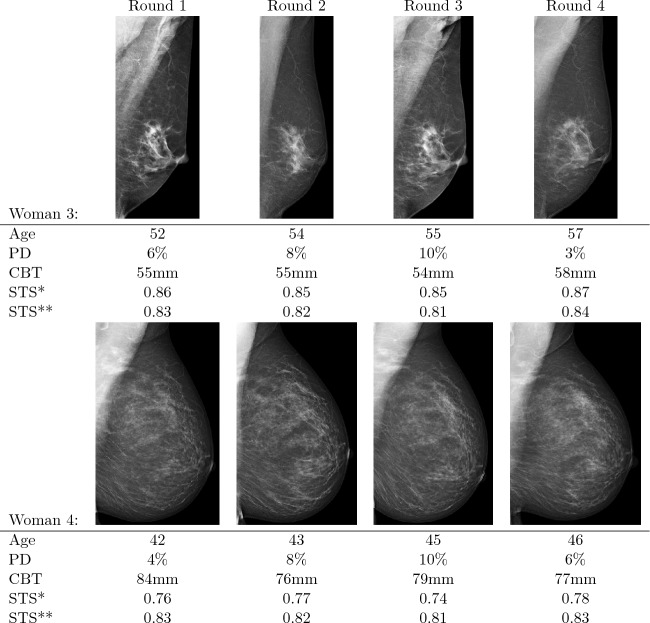
Example of two women’s sequential mammograms over 5 years, including age and the measured values of percent density (PD) and compressed breast thickness (CBT). *Estimated screening test sensitivity (STS) for a hypothetical 13 mm tumor when accounting for PD and CBT. **Estimated STS for a hypothetical 13 mm tumor when accounting only for PD.

To illustrate how CBT contributes to STS (how it complements PD), and how PD and CBT differ across screenings (for the same women), as well as across women, we present four examples (sets of images from four selected women). For each of the women, four mammograms were taken during the study period. Images from two women with high PD are shown in Fig. 4, and images from two women with low PD are displayed in Fig. 5. We present the woman’s age at the time each mammogram was taken, along with the measured PD and CBT values. We also display the estimated STS when including CBT (denoted STS* in the figures) and the estimated STS when not including CBT (denoted STS**) in the model-based STS measures. Both STS estimates are calculated for a hypothetical tumor diameter of 13 mm (the median in the study) and include PD in the estimate.

In discussions, below, of the images in Figs. 4 and 5, we use the notation i–j to refer to the image corresponding to Woman i and Round j. Woman 1 has high PD and CBT, and PD measurements reduce greatly over time from 57 to 24%. There is a single large increase in CBT in 1–4 from 79 to 90 mm. For all four images, the estimated STS is severely reduced (by around 0.20) when accounting for the high CBT. The average estimated STS difference when including CBT is 0.20 compared to not including CBT.

Woman 2 also has high PD, but has average CBT. Instead of decreasing over time, the PD measurements fluctuate between 39 and 50%. CBT values are between 54 and 61 mm. Also, for this woman, all estimated STS values are lower when CBT is accounted for; the estimated STS difference is on average 0.06.

Comparing images across women helps to highlight the impact of including CBT in the STS model/estimates. If we compare 1–4 to 2–2, which are the images with the highest estimated STS for the respective woman, we see that 1–4 has much lower PD (24% vs. 39%), and so, based solely on PD, has higher STS. However, when accounting for CBT, the estimated STS is not as low as that of 2–2. It is the high CBT of Woman 1 that drives the differences in STS estimates. Similarly, comparing 1–3 and 2–4, we see that despite having almost the same PD measurements, the estimated STS is significantly higher in 2–4 than in 1.3.

Woman 3 in Fig. 5 has relatively low PD and average CBT. The better separation of high and low STS means that the estimated STS is slightly higher when accounting for CBT. The mean estimated STS difference is −0.03.

Similarly, Woman 4 has low PD. However, the CBT values are significantly higher. Here we see that the estimated STS is instead noticeably lower when including CBT in the estimate. The estimated STS difference is 0.06 lower on average. The difference compared to Woman 3 is due to the high CBT. For example, images 3–3 and 4–3 have the same PD and the same estimated STS without CBT, but a 0.11 STS difference when accounting for CBT.

Lastly, we performed a sensitivity analysis of our choice to use the average measurements between the left and right side. We repeated the analysis where we used the measurements from the contralateral breast for the cases, and a randomly selected side for the censored women. The estimated coefficients for CP and EXP were smaller (scaled estimates of −0.08 and −0.16) but still not statistically significant (*p*-values 0.40 and 0.07). We also found that the coefficients for PD and CBT were only minimally impacted, with scaled estimates of −0.64 (95%CI −0.84, −0.44) and −0.27 (95%CI −0.45, −0.09).

## Discussion

We have investigated the role that PD and a number of screening parameters have on mammography screening sensitivity. We found that the STS was significantly reduced by having increased PD or increased CBT. While PD is well known to have a masking effect^14,16,35^, few studies have been done on the role of CBT in screening sensitivity. In the first part of a two-part study, Salvagnini et al.^36^ simulated masses and microcalcifications onto real mammograms—they used 130 subsets of images with each subset containing four images with different CBT, but matched by BI-RADS score^37^. Half of these images were kept lesion free. Radiologists, who were blinded to tumor/control status, attempted to identify the lesions. Examinations were performed with standard AEC settings. The detectability (as measured by area under the operating characteristic curve) was found to be lower for mammograms with higher CBT. Salvagnini et al.^36^ reported a free-response receiver operator characteristic of 0.802 for CBT under 30 mm and 0.553 for CBT over 60 mm. Our results, obtained from a population-based study, with real lesions, are consistent with these results.

As mentioned in the introduction, Moshina et al.^20^, and Holland et al.^19^ studied the association between compression force and/or pressure and detectability at mammography screening. They used the ratio-based definition of screening sensitivity. Both studies found that high CP reduced sensitivity and was associated with interval cancer. Hill et al.^21^ instead found the opposite association: Higher CP increased sensitivity. We can note that Fig. 2 in Holland et al. may suggest that too little compression also reduced the sensitivity (making the association follow an inverted U-shape with the highest sensitivity for CP between 0.93 and 1.08N/$\hbox {cm}^2$), though this non-linear relationship is not tested for in the study. In our study, we did not find any association with STS, but instead found the association to be between CBT and STS. The two other studies adjusted for TBV and mammographic density, but it would be interesting to see if the association they found with CP persists if also adjusting for CBT. It could be that CBT better represents the achieved compression.

We defined the STS as a screening-specific probability of detecting a BC given the tumor size at the time of screening. The approach involved modelling the latent onset and growth of BC tumors. While there is no substitute for multiple actual size measures, this approach should give valid inference results on a population level. Other studies have had a general lack of consideration for tumor size and growth rates when estimating screening sensitivity. The variation on mammography screening sensitivity that we study, is closely related to the statistical definition of sensitivity^13^, although with our definition, a positive “test” result is not based only on the radiologists assessment of the mammogram, but incorporates the outcome of any subsequent investigation, e.g. tomosynthesis, ultrasound, or biopsy that ultimately leads to diagnosis. Our STS is more aptly then defined as the probability that a screening leads to diagnosis, given the tumor size—and in this study—PD and CBT.

Comparing this definition to the standard definition of sensitivity in the context of mammography screening, we have previously mentioned that the standard definition assumes that all interval cancers were present at the last screening but missed, and that no cancer was present for more than one screening round. This puts unmotivated constraints on the natural history of the cancer. By using our approach to estimating the STS, all possibilities for the natural history are taken into account.

The standard definition is also dependent on the screening interval and the follow-up time^8^. Using this definition of screening sensitivity also leads to studies estimating different sensitivities for the prevalence screening (the first screening round in a study) and incidence screenings (subsequent screening rounds), with a significantly greater sensitivity for the prevalence screen^38^. This is due to the tumors being larger on average in the prevalence screen compared to the incidence screens^10^. With time and regular screening the estimated screening sensitivity will change. A major advantage of the type of model used in this study is that it automatically includes the difference between a prevalence screen and incidence screen by modelling latent tumors and incorporating individual screening histories. The estimated STS also does not depend on the screening interval or the follow-up time, given the concurrent latent tumor size distribution.

Screening using contrast-enhanced magnetic resonance imaging (MRI) is known to find more breast cancers than mammography screening, and to detect them sooner.^39–42^ The additional tumors detected at MRI are on average smaller than those detected at mammography. This highlights the importance of tumor size—something often neglected when reporting screening sensitivities. As can be seen in Fig. 3, the estimated median sensitivity for a 10 mm tumor was 45%, with the 5th percentile of sensitivity (i.e. for the most dense) was a mere 20%. It therefore follows naturally from our approach that the additional MRI-detected tumors have an especially low mammography sensitivity, and that MRI can detect such tumors sooner.

In a previous study^43^ we estimated the association between PD and STS to have a coefficient of −2.09 (95% CI −2.93, −1.26), which is noticeably smaller in magnitude than what we estimate in this study. One reason is the inclusion of the other screening variables. Another reason is that in the previous study we only had one measurement of PD per woman, taken from the screening where they were enrolled. In Fig. 2 we see how PD differs with age in our study. Some of the difference between estimates can probably be attributed to the fact that PD at the start of follow-up (when PD was measured in the previous study) was, on average, higher than at subsequent screenings. Having multiple measurements of PD (and by extension the other screening variables) has improved the estimates.

For Woman 1 in Fig. 4, the 4th image had an estimated PD of 24%, which was perhaps lower than was motivated by its appearance. That image also had a larger CBT than the other three by 10 mm. This could have caused insufficient spreading out of the breast tissue, which in turn led to a relatively low contrast between dense and non-dense tissue. As a result, the PD would be underestimated. By accounting for the raised CBT, the estimated sensitivity is lowered to be closer to that of the previous images, thus partially offsetting the underestimated PD.

It is also important to note that the association between compressed breast thickness and mammographic screening sensitivity/STS that we observed, after adjustment for PD, is based on an area-based density measure. The relationships of both area and volumetric PD with sensitivity have been extensively studied^44–46^. If volumetric PD measurement is more consistent across various levels of compression than area PD, it is possible that the influence of thickness might be lower.

There are of course limitations to our modelling approach. Here we chose to model the STS as a logistic function of tumor diameter. While this is a function with desirable properties, it is a choice that restricts the shape of the STS. Wang et al.^47^ tried to estimate the STS without parametric assumptions. By assuming exponential tumor growth with the same constant tumor volume doubling time for all tumors, their results suggested that the logistic function underestimates the STS for small tumors, and that the STS seemingly approaches 1 too quickly. Alternative functions might improve model fit, but for this study we do not foresee that the associations between the screening variables and the STS are significantly affected.

In recent years, there has a great deal of interest in improving ways to screen women for which breast cancer risk is high and mammographic sensitivity is low^48,49^. It has been suggested that women’s screening intervals should be increased based on density^50^, or for high density women to be offered additional screening modalities^46,51^. The motivation is based on a dual effect of an increased BC risk, as well an increased risk of masking at mammography. While density remains the dominant factor of risk and sensitivity, the results of this study suggest that additional imaging parameters might be considered in the decision-making.

With regard to this, improving understanding of mammographic sensitivity is important, as are approaches to improving sensitivity. In part 2 of Salvagnini et al.’s study of compressed breast thickness on lesion detectability^36^, a new AEC setup was proposed and studied, to see if lesion detectability can be improved using larger breast thickness. The results from part 1 of their study, based on simulated lesions, along with our results, based on real images/lesions in a large population-based study, provide motivation for such endeavours.

## Conclusions

The true sensitivity of mammography, defined as the probability that an examination leads to a positive result if a tumour is present in the breast, is associated with compressed breast thickness after accounting for mammographic density and tumour size. This can be used to guide studies of setups aimed at improving lesion detection. These results can help motivate further research into mammography screening settings. Our results suggest that other screening parameters than mammographic density—specifically the compressed breast thickness—should be considered when assigning alternative screening modalities, and when considering personalized screening in the future.

### Supplementary Information

Supplementary Information.

## Data Availability

The data that support the findings of this study are available from karmastudy.org but restrictions apply to the availability of these data, which were used under license for the current study, and so are not publicly available. Data are however available from the authors upon reasonable request and with permission of karmastudy.org. For request, contact Keith Humphreys (keith.humphreys@ki.se).

## References

[1] DeSantis CE, Ma J, Gaudet MM, Newman LA, Miller KD, Goding Sauer A (2019). Breast cancer statistics, 2019. CA Cancer J. Clin..

[2] Bray F, Ferlay J, Soerjomataram I, Siegel RL, Torre LA, Jemal A (2018). Global cancer statistics 2018: GLOBOCAN estimates of incidence and mortality worldwide for 36 cancers in 185 countries. CA Cancer J. Clin..

[3] Altobelli E, Rapacchietta L, Angeletti PM, Barbante L, Profeta FV, Fagnano R (2015). Breast cancer screening programmes across the who European region: Differences among countries based on national income level. Int. J. Environ. Res. Public Health.

[4] Olsson S, Andersson I, Karlberg I, Bjurstam N, Frodis E, Håkansson S (2000). Implementation of service screening with mammography in Sweden: From pilot study to nationwide programme. J. Med. Screen..

[5] Korhonen KE, Zuckerman SP, Weinstein SP, Tobey J, Birnbaum JA, McDonald ES (2021). Breast MRI: False-negative results and missed opportunities. Radiographics.

[6] Théberge I, Guertin MH, Vandal N, Daigle JM, Dufresne MP, Wadden N (2018). Clinical image quality and sensitivity in an organized mammography screening program. Can. Assoc. Radiol. J..

[7] Lehman CD, Arao RF, Sprague BL, Lee JM, Buist DS, Kerlikowske K (2017). National performance benchmarks for modern screening digital mammography: Update from the Breast Cancer Surveillance Consortium. Radiology.

[8] Hofvind S, Geller B, Skelly J, Vacek P (2012). Sensitivity and specificity of mammographic screening as practised in Vermont and Norway. Br. J. Radiol..

[9] Buist DS, Porter PL, Lehman C, Taplin SH, White E (2004). Factors contributing to mammography failure in women aged 40–49 years. J. Natl Cancer Inst..

[10] Hollingsworth AB (2019). Redefining the sensitivity of screening mammography: A review. Am. J. Surg..

[11] Weedon-Fekjær H, Tretli S, Aalen OO (2010). Estimating screening test sensitivity and tumour progression using tumour size and time since previous screening. Stat. Methods Med. Res..

[12] Abrahamsson L, Humphreys K (2016). A statistical model of breast cancer tumour growth with estimation of screening sensitivity as a function of mammographic density. Stat. Methods Med. Res..

[13] Trevethan R (2017). Sensitivity, specificity, and predictive values: Foundations, pliabilities, and pitfalls in research and practice. Front. Public Health.

[14] Lynge E, Vejborg I, Andersen Z, von Euler-Chelpin M, Napolitano G (2019). Mammographic density and screening sensitivity, breast cancer incidence and associated risk factors in Danish breast cancer screening. J. Clin. Med..

[15] Mandelson MT, Oestreicher N, Porter PL, White D, Finder CA, Taplin SH (2000). Breast density as a predictor of mammographic detection: Comparison of interval-and screen-detected cancers. J. Natl Cancer Inst..

[16] Chiu SYH, Duffy S, Yen AMF, Tabár L, Smith RA, Chen HH (2010). Effect of baseline breast density on breast cancer incidence, stage, mortality, and screening parameters: 25-year follow-up of a Swedish mammographic screening. Cancer Epidemiol. Biomark. Prev..

[17] Ekpo EU, Alakhras M, Brennan P (2018). Errors in mammography cannot be solved through technology alone. Asian Pac. J. Cancer Prev..

[18] Fausto A, Lopes M, de Sousa M, Furquim T, Moi A, Velasco F (2017). Optimization of image quality and dose in digital mammography. J. Digit. Imaging.

[19] Holland K, Sechopoulos I, Mann RM, Den Heeten GJ, van Gils CH, Karssemeijer N (2017). Influence of breast compression pressure on the performance of population-based mammography screening. Breast Cancer Res..

[20] Moshina N, Sebuødegård S, Hofvind S (2017). Is breast compression associated with breast cancer detection and other early performance measures in a population-based breast cancer screening program?. Breast Cancer Res. Treat..

[21] Hill, M. L., Martis, L., Halling-Brown, M., Highnam, R. P. & Chan, A. Mammographic compression pressure as a predictor of interval cancer. In: *16th International Workshop on Breast Imaging (IWBI2022)* vol. 1228612, 28 (SPIE, 2022).

[22] Jeukens C, Van Dijk T, Berben C, Wildberger J, Lobbes M (2019). Evaluation of pressure-controlled mammography compression paddles with respect to force-controlled compression paddles in clinical practice. Eur. Radiol..

[23] de Groot JE, Branderhorst W, Grimbergen CA, den Heeten GJ, Broeders MJM (2015). Towards personalized compression in mammography: A comparison study between pressure- and force-standardization. Eur. J. Radiol..

[24] Kelemen LE, Pankratz VS, Sellers TA, Brandt KR, Wang A, Janney C (2008). Age-specific trends in mammographic density: The Minnesota breast cancer family study. Am. J. Epidemiol..

[25] McCormack VA, Perry NM, Vinnicombe SJ, dos Santos SI (2010). Changes and tracking of mammographic density in relation to Pike’s model of breast tissue aging: A UK longitudinal study. Int. J. Cancer.

[26] Keen JD, Keen JE (2008). How does age affect baseline screening mammography performance measures? A decision model. BMC Med. Inform. Decis. Mak..

[27] Holland K, van Zelst J, den Heeten GJ, Imhof-Tas M, Mann RM, van Gils CH (2016). Consistency of breast density categories in serial screening mammograms: A comparison between automated and human assessment. The Breast.

[28] Kim WH, Moon WK, Kim SM, Yi A, Chang JM, Koo HR (2013). Variability of breast density assessment in short-term reimaging with digital mammography. Eur. J. Radiol..

[29] Alonzo-Proulx O, Mawdsley GE, Patrie JT, Yaffe MJ, Harvey JA (2015). Reliability of automated breast density measurements. Radiology.

[30] Sprague BL, Conant EF, Onega T, Elisabeth F, Beaber MPG, Herschorn SD, Lehman CD (2016). Variation in mammographic breast density assessments among radiologists in clinical practice. Ann. Intern. Med..

[31] Gabrielson M, Eriksson M, Hammarström M, Borgquist S, Leifland K (2017). Cohort profile: The Karolinska mammography project for risk prediction of breast cancer (KARMA). Int. J. Epidemiol..

[32] Eriksson M, Czene K, Pawitan Y, Leifland K, Darabi H, Hall P (2017). A clinical model for identifying the short-term risk of breast cancer. Breast Cancer Res..

[33] Strandberg JR, Humphreys K (2019). Statistical models of tumour onset and growth for modern breast cancer screening cohorts. Math. Biosci..

[34] Moolgavkar SH, Luebeck G (1990). Two-event model for carcinogenesis: Biological, mathematical, and statistical considerations. Risk Anal..

[35] Mandelson MT, Oestreicher N, Porter PL, White D, Finder CA (2000). Breast density as a predictor of mammographic detection: Comparison of interval- and screen-detected cancers. JNCI J. Natl. Cancer Inst..

[36] Salvagnini E, Bosmans H, Van Ongeval C, Van Steen A, Michielsen K, Cockmartin L (2016). Impact of compressed breast thickness and dose on lesion detectability in digital mammography: FROC study with simulated lesions in real mammograms. Med. Phys..

[37] D’Orsi CJ, Sickles EA, Mendelson EB, Morris EA (2013). ACR BI-RADS Atlas, Breast Imaging Reporting and Data System.

[38] Humphrey LL, Helfand M, Chan BKS, Woolf SH (2002). Breast cancer screening: A summary of the evidence for the U.S. preventive services task force. Ann. Intern. Med..

[39] Lee JM, Ichikawa L, Valencia E, Miglioretti DL, Wernli K, Buist DSM (2017). Performance benchmarks for screening Breast MR Imaging in community practice. Radiology.

[40] Saadatmand S, Geuzinge HA, Rutgers EJT, Mann RM, de Roy van Zuidewijn DBW, Zonderland HM (2019). MRI versus mammography for breast cancer screening in women with familial risk (FaMRIsc): A multicentre, randomised, controlled trial. Lancet Oncol..

[41] Mann RM, Kuhl CK, Moy L (2019). Contrast-enhanced MRI for breast cancer screening. J. Magn. Reson. Imaging.

[42] Kornecki A (2022). Current status of contrast enhanced mammography: A comprehensive review. Can. Assoc. Radiol. J..

[43] Strandberg R, Czene K, Eriksson M, Hall P, Humphreys K (2022). Estimating distributions of breast cancer onset and growth in a Swedish mammography screening cohort. Cancer Epidemiol. Biomark. Prevent..

[44] Destounis S, Johnston L, Highnam R, Arieno A, Morgan R, Chan A (2016). Using volumetric breast density to quantify the potential masking risk of mammographic density. Am. J. Roentgenol..

[45] Wanders JOP, Holland K, Karssemeijer N, Peeters PHM, Veldhuis WB, Mann RM (2017). The effect of volumetric breast density on the risk of screen-detected and interval breast cancers: A cohort study. Breast Cancer Res..

[46] Larsen M, Lynge E, Lee CI, Lång K, Hofvind S (2023). Mammographic density and interval cancers in mammographic screening: Moving towards more personalized screening. Breast.

[47] Wang J, Gottschal P, Ding L, van Veldhuizen DW, Lu W, Houssami N (2020). Mammographic sensitivity as a function of tumor size: A novel estimation based on population-based screening data. Breast.

[48] Schousboe JT, Kerlikowske K, Loh A, Cummings SR (2011). Personalizing mammography by breast density and other risk factors for breast cancer: Analysis of health benefits and cost-effectiveness. Ann. Intern. Med..

[49] Román M, Sala M, Domingo L, Posso M, Louro J, Castells X (2019). Personalized breast cancer screening strategies: A systematic review and quality assessment. PLoS ONE.

[50] TBST study group. *Tailored Screening for Breast Cancer in Premenopausal Women (TBST)* (Bethesda (MD): National Library of Medicine (US), ClinicalTrialsgov. 2015) Report No: NCT02619123. Available from: https://clinicaltrials.gov/ct2/show/NCT0261912329.

[51] Gray E, Donten A, Karssemeijer N, van Gils C, Evans DG, Astley S (2017). Evaluation of a stratified national breast screening program in the United Kingdom: An early model-based cost-effectiveness analysis. Value Health.

