# Supplementary material for: Influence of mammographic density and compressed breast thickness on true mammographic sensitivity: a cohort study
Source: Sci Rep. 2023 Aug 30;13:14194. doi: 10.1038/s41598-023-41356-2 (PMC10468499; doi:10.1038/s41598-023-41356-2)
Supplement: Supplementary file 1 — Supplementary Information. [file 41598_2023_41356_MOESM1_ESM.pdf]

Supplementary Material

Here we describe all four components of the natural history model in more detail. We the describe how the model is calibrated using only data from breast cancer diagnosis on age, tumor size, mode of detection, and screening history.

Sub-model 1: Age at onset

We start with the Moolgavkar-Venson-Knudson (MVK) two-stage model of carcinogenesis [1, 2] to describe the time from birth to the age at onset T of an invasive breast cancer tumor. The survival function for this model is given by

$$G_T(t) = P(T > t) = \left[\frac{(B-A)e^{Bt}}{Be^{(B-A)t} - A}\right]^{\delta},$$

with the parameters $A < 0$ and $B, \delta > 0$. The corresponding probability density function (PDF) is

$$f_T(t) = -\frac{\mathrm{d}G_T(t)}{\mathrm{d}t} = \delta AB(B-A)^{\delta} \frac{e^{\delta Bt}\left(1 - e^{(B-A)t}\right)}{\left(Be^{(B-A)t} - A\right)^{\delta+1}}.$$

For practical reasons, we assume that the tumor is a sphere with a starting diameter of 0.5mm at age $T = t$. From that point, we assume that the tumor will grow deterministically, and will be detectable with non-zero probability.

Sub-model 2: Tumor growth

Once onset occurs, we assume that the tumor is a sphere that grows exponentially. Given that onset occurred at age $T = t$, and given an inverse growth rate r, the tumor volume (mm^3) and diameter (mm) at age $x > t$ are given by

$$V(x) = v_0 e^{\frac{x-t}{r}} \quad \text{and} \quad D(x) = d_0 e^{\frac{x-t}{3r}},$$

where $d_0 = 0.5$(mm), and v_0 is the volume of the sphere with diameter d_0, i.e. the starting tumor diameter and volume, respectively.

To represent the heterogeneity in the tumor growth rates between tumors, we assume that the

inverse growth rate of each tumor is drawn from a gamma-distributed random variable $R = r$, with PDF

$$f_R(r) = \frac{(\mu\phi)^{-1/\phi}}{\Gamma(1/\phi)} r^{\frac{1}{\phi}-1} \exp\left(-\frac{r}{\mu\phi}\right),$$

for the parameters $\mu\phi > 0$, where $E[R] = \mu$ and $Var(R) = \phi\mu^2$. While the individual inverse growth rates are not observed, the available data can be used to estimate the parameters μ and ϕ, and thus the inverse growth rate distribution of the study population.

Sub-model 3: Symptomatic detection

As the tumor progresses in size, the breast cancer has an increasing risk of displaying symptoms, such as a palpable lump, redness, or swelling. These symptoms then lead to detection of the cancer. We assume that the continuous hazard rate of symptomatic detection at age u is proportional to the latent tumor volume $V(u)$, i.e. for the age at symptomatic detection U:

$$P(U \in (u, u + \Delta u] | U > u) = \eta V(u) \Delta u + o(\Delta u),$$

for the parameter $\eta > 0$. In this study, we let η depend on TBV through

$$\log(\eta) = \eta_0 + \eta_1 [TBV].$$

Since TBV is only measured at screening, we use each woman's average TBV across screenings as a constant effect on the symptomatic detection rate.

Sub-model 4: Screen-detection

If a woman attends mammography screening before a tumor is symptomatically detected, there is opportunity to detect it early. We assume that the screening test sensitivity (STS) for the mammography screening undertaken at age ω follows a logistic function of the latent tumor diameter $D(x)$, i.e.

$$STS(\omega) = \text{logit}\left(\beta_0 + \beta_s D(\omega)\right) = \frac{\exp\left(\beta_0 + \beta_s \cdot D(\omega)\right)}{1 + \exp\left(\beta_0 + \beta_s \cdot D(\omega)\right)},$$

for parameters β_0, β_s. The STS can be extended to depend on other factors than just size. In this study, we include the possible dependence on PD, CBT, EXP, CP. The STS function is then

$$STS(\omega) = \text{logit}\left(\beta_0 + \beta_s D(\omega) + \beta_1[PD] + \beta_2[CBT] + \beta_3[EXP] + \beta_4[CP]\right).$$

Over her lifetime (and up to her end of follow-up), a woman will be invited to attend mammography screening at certain ages, each of which she can choose to attend or not. This creates an individual screening history for each woman in the study, consisting of the ages when screened, and the results of each screening (positive or negative). For most women, this will be a sequence of negative screenings. Only the screen-detected cases will have one positive result—their final screening. If a woman attended screening at ages $\tau_1, \tau_2, \ldots, \tau_k$, the probability of her screening history is

$$P(\text{History}) = \begin{cases} STS(\omega_k) \displaystyle\prod_{j=1}^{k-1} \left(1 - STS^*(\omega_j)\right), & \text{if screen-detected,} \\ \displaystyle\prod_{j=1}^{k} \left(1 - STS^*(\omega_j)\right), & \text{otherwise,} \end{cases}$$

where $STS^*(\omega_j)$ is the STS when the tumor size at age ω_j is unknown. Since tumor size is only measured at diagnosis, then—for all but the screen-detected cases—these latent tumor sizes must be inferred by combining all four sub-models. For screen-detected cases, tumor sizes at all previous negative screenings need to be inferred in the same way.

This process of screen-detection becomes a competing risk to symptomatic detection, where only the first detection mode is observed. A cancer which is symptomatically detected between two mammography screenings is commonly referred to as an interval cancer.

Likelihood function

The four sub-models are combined into an individual timeline for each woman in the study. This constructed timeline is used to calculate the likelihood of the observed outcome. Thus, the unknown parameters in the sub-models can be estimated. The formulas for these individual likelihood contributions have been derived and presented previously [3, 4]. The observed outcome for each BC case in the study is the age at detection, tumor size at detection, and the mode of detection. The

likelihood of being detected at age x with a tumor of volume v is

$$L_{case}(x,v) = C \int_0^x \frac{(x-t)\exp\left[-\eta(v-v_0)\frac{x-t}{\ln(v/v_0)}\right] f_R\left(\frac{x-t}{\ln(v/v_0)}\right) f_T(t)}{\prod\limits_{j|t\leq\omega_j\leq x}\left(1+\exp\left(\beta_0+\beta_s d_0 (v/v_0)^{\frac{1}{3}(\omega_j-t)/(x-t)}+\ldots\right)\right)} \mathrm{d}t$$

where

$$C = \begin{cases} \frac{\eta}{\ln^2(v/v_0)} & \text{, if symptomatic} \\ \frac{\exp(\beta_0+\beta_s d+\ldots)}{v\ln^2(v/v_0)} & \text{, if screen-detected} \end{cases}.$$

By marginalizing over the age at onset, we can use the start point (age at onset of a 0.5mm tumor) and end point (detection at age x with a $v$mm^3 tumor) to calculate the probability of the specific observed outcome (mode, age, and tumor size). For censored individuals we do not have information on the final tumor size (nor of its existence). We therefore need to compound over the inverse growth rate R as well. Then the same idea lets us calculate the likelihood of *not* being detected by either mode up to end of follow-up:

$$L_{cens}(x) = G_T(x) + \int_0^x \int_0^\infty \frac{\exp\left[-\eta r(v_0 e^{(x-t)/r} - v_0)\right] f_R(r) f_T(t)}{\prod\limits_{j|t\leq\omega_j\leq x}\left(1+\exp\left(\beta_0+\beta_s d_0 e^{\frac{1}{3}(\omega_j-t)/r}+\ldots\right)\right)} \mathrm{d}r\mathrm{d}t.$$

For the derivations of these likelihood functions, and how they are adjusted for left truncation, see [3].

Each individual's likelihood contribution is then combined into a total log-likelihood. For N study participants, the total log-likelihood is

$$logL = \sum_{i=1}^{N} \ln(L_*(x_i[,v_i]).$$

This total log-likelihood is then maximized with respect to the parameters of the four submodels. These parameters are $A, B, \delta, \mu, \phi, \eta_0, \beta_0, \beta_s$, in addition to parameters for any additional covariates of interest.

References

[1] Moolgavkar SH, Knudson AG. Mutation and cancer: A model for human carcinogenesis. Journal of the National Cancer Institute. 1981;66(6):1037-52.

[2] Moolgavkar SH, Luebeck G. Two-Event Model for Carcinogenesis: Biological, Mathematical, and Statistical Considerations. Risk Analysis. 1990;10(2):323-41.

[3] Strandberg JR, Humphreys K. Statistical models of tumour onset and growth for modern breast cancer screening cohorts. Mathematical Biosciences. 2019;318:108270.

[4] Strandberg R, Czene K, Eriksson M, Hall P, Humphreys K. Estimating Distributions of Breast Cancer Onset and Growth in a Swedish Mammography Screening Cohort. Cancer Epidemiology Biomarkers and Prevention. 2022.
